# Annotation of gene sequence and protein structure of brinjal EDS1

**DOI:** 10.6026/97320630013054

**Published:** 2017-03-02

**Authors:** Soumya Sharma, Sarika Jaiswal, Sunil Archak

**Affiliations:** 1 Division of Bioinformatics, ICAR-IARI, Pusa Campus, New Delhi, India;; 2 Centre for Agricultural Bioinformatics, ICAR-IASRI, Pusa Campus, New Delhi, India;; 3 Division of Genomic Resources, IACR-NBPGR, Pusa Campus, New Delhi, India;

**Keywords:** brinjal, EDS1, phylogenetic analysis, structure prediction

## Abstract

Enhanced Disease Susceptibility1 (EDS1) is a nucleo-cytoplasmic protein, known to be a key regulator of plant basal defense and effector-triggered immunity. Sequence of a single copy brinjal EDS1 gene (SmEDS1) was mined from draft brinjal genome assembly. The extracted sequence was found to be incomplete and polished with the help of transcriptome sequence data. Full-length SmEDS1 gene is 4.5kb long having three exons that coded for 1.8kb mRNA. SmEDS1 protein is a 602 amino acid long protein consisting of Lipase3 and EP domain regions. Predicted tertiary structure of SmEDS1 using homology modelling had a mass of 68.8kD and was made of 10 strands, 26 alpha helices, five 310 helices and 43 beta turns. Phylogenetic analysis based on protein sequence grouped the species in clades defined by botanical family suggesting that EDS1 protein has evolved through the speciation process. Phylogenetic tree based on EDS1 structures grouped Solanum species of American origin (tomato, wild tomato and potato) together but brinjal EDS1 (Asiatic origin) occupied a unique position. In silico information generated in this study is expected to be the first step toward cloning and expression analysis of SmEDS1 gene.

## Background

Effector triggered immunity (ETI), responsible for disease resistance in plants [1], is activated by a series of signaling events resulting from alteration of R protein interactions. However, only a small number of genes have been identified through genetic studies which are considered to encode proteins acting downstream of R proteins for defense signaling [2]. Among these, nucleo-cytoplasmic Enhanced Disease Susceptibility1 (EDS1) has been recognized as the key regulator of ETI initiated via TIR-NBLRR class of R proteins and hence plant basal defense. Studies in Arabidopsis have shown that EDS1 family proteins constitute a regulatory center for numerous stress-signaling pathways in plants including PAD4-associated basal defense, ETI stimulated through TIR-NB-LRR, ROS-associated regulation of cell death, salicylic acid mediated systemic acquired resistance, insect resistance, etc.

Brinjal (Solanum melongena L.) is an important solanaceous vegetable crop of Asiatic origin. Production of brinjal is affected by diseases (wilt, blight, rot, etc.), nematodes and insects. As a result, identification genes imparting biotic stress resistance in brinjal is the main focus for varietal development and the development of genetically modified organisms. EDS1 could be an ideal target as it regulates both basal as well as effector triggered defense pathways. As EDS1 is the key node of defense regulatory system that functions at a very primary level, its modulation using CRISPR/Cas9 [3] offers enormous opportunities for developing resistant varieties. Brinjal, being an atypical Solanum, has remained under-investigated with 133 genes in NCBI gene database against 30K of tomato, 33K potato and 72K tobacco genes [4]. In spite of the availability of a scaffold-level genome sequence [5], brinjal remains a “genomic orphan species” [6]. One of the possible reasons for lesser number of studies on brinjal could be the fact that it is not a popular vegetable outside Asia [7].

In the present study we report identification and characterization of Solanum melongena EDS1 gene (SmEDS1) and propose its protein structure. We also present molecular phylogenetic analysis based on EDS1 protein sequences and structures among plant species. In silico information generated in this study is expected to be the first step toward cloning and expression analysis of SmEDS1.

## Methodology

### Identification SmEDS1

Brinjal genome [5] was searched for EDS1 homologue using Arabidopsis thaliana EDS1 (AAD20950.1) as query sequence. The best hit obtained in the eggplant genome database [8] was used to search brinjal transcriptome generated in the laboratory (unpublished) to find the SmEDS1 transcript. Promoter prediction was done using the PlantProm DB and TSSP [9]. The analysis of the predicted promoter sequence was carried out using PlantCARE database [10]. Longest open reading frame from the transcript was selected as putative coding sequence and the resultant protein encoded as SmEDS1. To identify the protein family, domain and functional sites the predicted SmEDS1 protein sequence was submitted to InterPro protein families database, Pfam; conserved domain database (CDD), and Evolutionary classification of protein domains (ECOD) database.

### Protein 3D-structure modeling

SmEDS1 (602 amino acid) protein sequence was subjected to protein structure modelling using Swiss-model, a homology modelling web server [11]. Structure assessment and quality check [12] was carried out at Swiss-model server using Procheck [13]. Comparative modelling software, Modeller release 9.16 [14], was employed to build homology structures of Brinjal EDS1 based on AtEDS1 structure (PDB id 4NFU). The best model was selected based on Stereochemistry check by PROCHECK [13]. The selected structure was then subjected to energy minimization for refining the model using Hmod3DMM (energy minimization program by molecular mechanics) at Softberry [9]. The structure finally generated was designated as SmEDS1.pdb.

### Reconstruction of phylogeny based on sequence and structure

All available plant EDS1 sequences were collated by querying NCBI protein database, EggNOG database [15] and Ensemble plants [16]. After removing PAD4 and SAG101 protein sequences, sequences from 48 plant species were retained for analysis. Multiple sequence alignment of EDS1 sequences was carried out using ClustalW [17] and phylogenetic tree was generated using MEGA version 6.0 [18] using Neighbor Joining method with Poisson distribution, pairwise deletion, and bootstrap values of 1000 replications [19].

One representative from each clade of sequence-based Neighbor Joining tree was taken for 3D structure-based phylogenetic analysis based on structural comparison. Structure of each of the selected protein sequence (other Solanaceae species [Nicotiana tabacum, Solanum lycopersicon, Solanum tuberosum, Solanum pennellii, Capsicum annum], Brassica oleracea var. oleracea, Frageria vesca, Glycine max, Gossypium ramondii, Oryza sativa indica, and Vitis vinifera) was predicted by homology modelling. Structural alignment of the modelled structures and reconstruction of phylogeny was carried out using Multiseq [20].

## Result and discussion

### Architecture of SmEDS1

**Figure 1:**
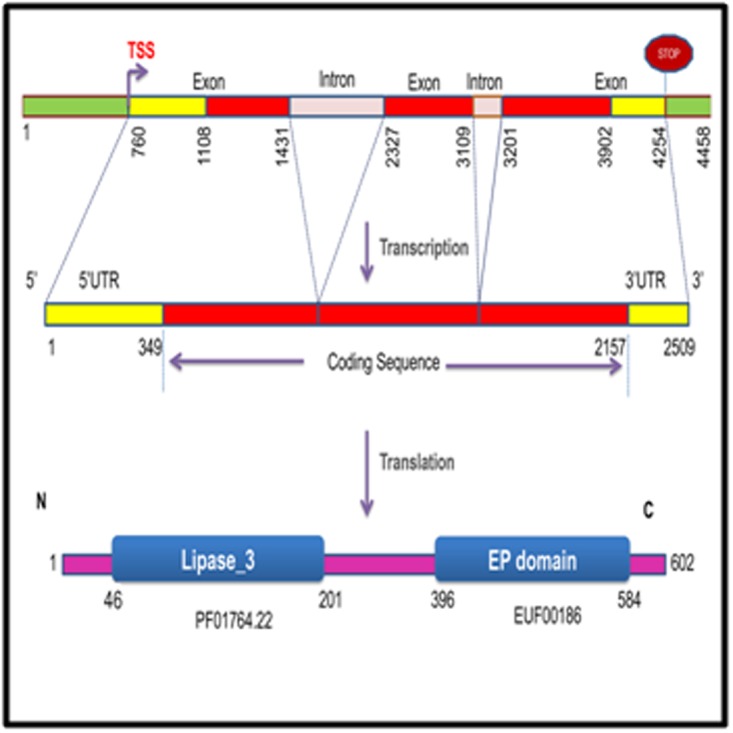
Architecture of brinjal EDS1 gene and protein. SmEDS1 comprised three exons (red and yellow) and two introns (grey) with a transcription start site (TSS) at position 760. The gene codes for a 2509 base mRNA having 5’ and 3’UTR regions (yellow). Protein coding region (red) consisted of 1809 nucleotides with 42.1% GC content. The SmEDS1 protein was made up of 602 amino acids with typical features of EDS1 such as N-terminal Lipase_3 domain (protein family Pfam ID mentioned below the box) and C-terminal EP domain (evolutionary classification of protein domain structures, ECOD database ID mentioned below box).

Mining the brinjal genome database using tblastn algorithm with AtEDS1 as the query sequence showed a homologue on the contig Sme2.5_09498.1 (e-value 1e-67). Ab initio gene prediction by FGENESH resulted in the gene coding for 486 amino acids whereas homology based gene prediction by FGENESH+ [9] showed gene coding for 529 amino acids. Gene sequence was polished using brinjal transcriptome sequence data (unpublished) available in the laboratory (Division of Genomic resources NBPGR, New Delhi) to extract full-length transcript. Longest open reading frame obtained by translating the transcript in all six possible frames using ExPASy-Translate tool coded for a 602 amino acid long protein. This step showed that SmEDS1 sequence mined from brinjal genome sequence was short by at least 73 amino acid residues and our analysis resulted in the fulllength SmEDS1 sequence. The resultant gene sequence was utilized to elucidate SmEDS1 gene structure (Figure 1). SmEDS1 comprised three exons and coded for a 2509 base mRNA having 5’ and 3’UTR regions. Protein coding region consisted of 1809 nucleotides with 42.1% GC content. InterPro scan resulted in an N-terminal alpha/beta hydrolase fold (IPR029058).

An N-terminal lipase_3 (PF01764.22) was predicted in Pfam result. CDD results predicted Esterase-lipase superfamily domain in range 44-217. Search by protein sequence using blast algorithm in ECOD database displayed hit in N-terminal Lipase_3 domain and C-terminal EP domain. All these features were typical to EDS1 proteins. The promoter sequence analysis using the PlantCARE database indicated toward the presence of ABRE element (ABscisic acid Response Element), Box-4, G-box CATTmotif and sp1 (light-responsiveness), TC-rich repeats (defenseand stress-response) and HSE element (heat stress response).

### Tertiary structure of SmEDS1 protein

**Figure 2:**
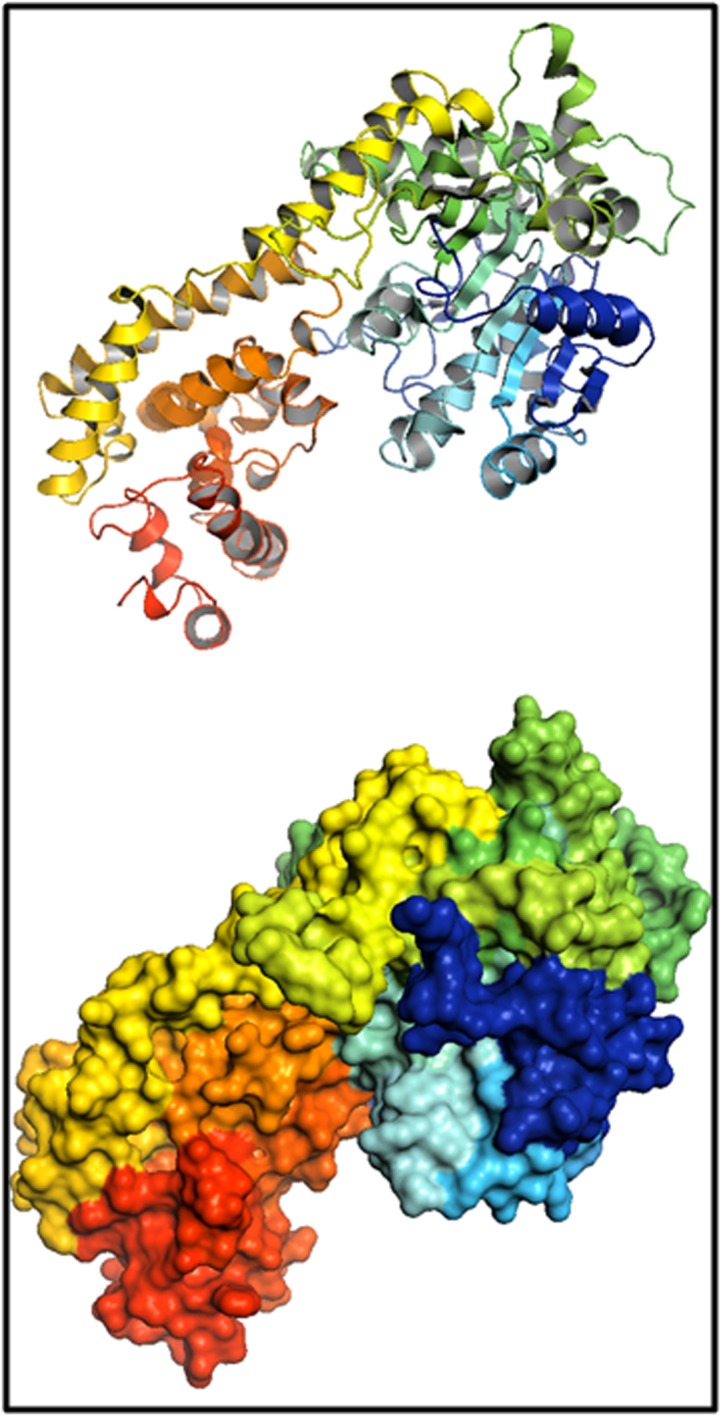
Structure of SmEDS1 protein depicted as ribbons (top) and surface model (bottom). The predicted structure has a mass of 68.78 kD and was made of 10 strands, 26 alpha helices, five 310 helices and 43 beta turns.

**Figure 3:**
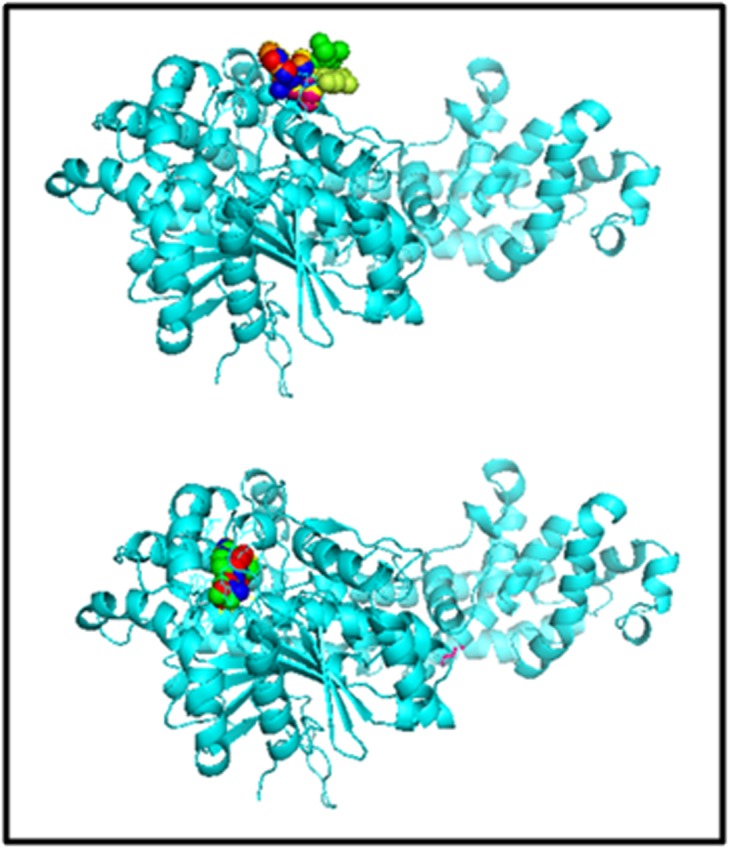
Potential binding sites of SmEDS1. 3DLigandsite Server predicted N-acetyl-D-glucosamine ligand with highest average MAMMOTH (top), whereas Ferulic acid was predicted with highest C-score by COACH meta-server (bottom).

Tertiary structure of SmEDS1 was modelled using AtEDS1 crystal structure (4NFU). The predicted structure was made of 10 strands, 26 alpha helices, five 310 helices and 43 beta turns (Figure 2). The brinjal EDS1 protein mass calculated from structure in PyMOL was 68.78 kD. Ramachandran’s statistics confirmed occurrence of 92.4% residues in most favored regions and 5.1% in additional allowed regions. 3DLigandsite Server predicted Nacetyl- D-glucosamine (NAG) ligand with highest average MAMMOTH, whereas FER (Ferulic acid) was predicted with highest C-score by COACH meta-server (Figure 3).

### Molecular phylogeny based on EDS1

**Figure 4:**
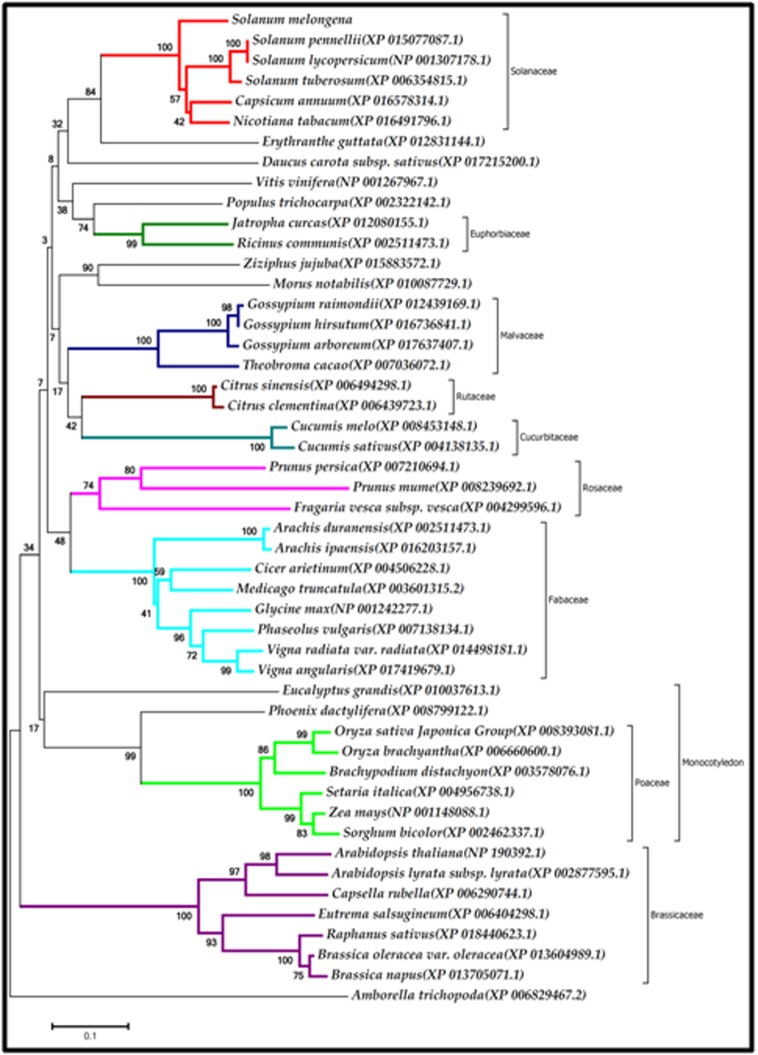
Phylogenetic analysis of EDS1 protein sequences representing 49 plant species grouped them according to the botanical family. NCBI sequence IDs are given in parentheses. Each clade is colored and designated with botanical family of the members. Bootstrap values are shown on the neighbor-joining tree.

Phylogenetic analysis of EDS1 protein sequences representing 49 plant species using neighbor-joining tree grouped the species belonging to the same family together in a single clade (Figure 4). Such a tree structure indicated that EDS1 protein sequences have evolved along the process of speciation. In all EDS1 orthologs, a serine-aspartate-histidine catalytic triad and a turn-causing GxSxG-motif were strictly conserved in SmEDS1 as among other EDS1 orthologues. Two citrus species and cocoa were exceptions with serine embedded in GxSxA motif.

**Figure 5:**
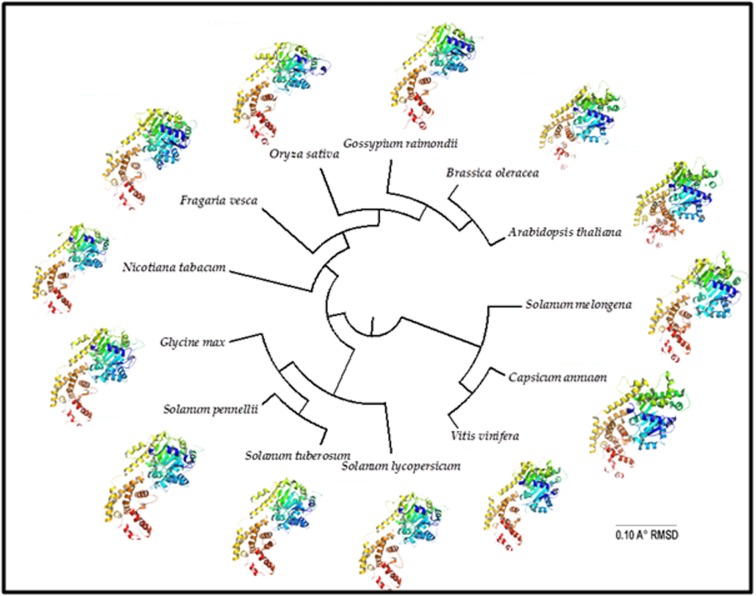
Phylogenetic tree of EDS1 structures of 13 plant species constructed based on Root Mean Square Deviation values. The structures, shown against species, were modelled using AtEDS1 crystal structure (4NFU). Based on EDS1 structure, brinjal occupied a distinct cluster whereas other solanaceous members— tomato, wild tomato and potato were grouped together.

A common measure of structural similarity between two homologous protein structures is root mean square deviation (RMSD) of topologically equivalent C-alpha atoms and used to for phylogenetic analysis [21]. The topology and grouping of species in the phylogenetic tree (Figure 5) constructed based on structural alignment (RMSD) was different from the one observed in phylogenetic tree generated based on sequence alignment. Brinjal occupied a distinct cluster with capsicum whereas other three Solanum species — tomato, potato and S. pennelli — were grouped together.

## Conclusion

Identifying, annotating and characterizing EDS1 protein in crops like brinjal, which is seriously affected by biotic stress, can provide a novel way in resistance breeding. Availability of scaffold-level genome sequence and new possibilities via geneediting technologies motivated the identification and characterization of EDS1 in brinjal. Amino acid sequence based phylogenetic grouping of plant EDS1 proteins exhibited congruence with taxonomy. This observation indicates the evolutionary and hence functional significance of EDS1 in plants. 3D structure-based phylogenetic relationships on the other hand revealed no such analogy with genera or species. In silico analysis has revealed conservation of sequence motifs and structures to a large extent among plant EDS1 proteins. SmEDS1 is surmised as an ideal candidate for genetic engineering experiments.
